# Don’t look now! Emotion-induced blindness: The interplay between emotion and attention

**DOI:** 10.3758/s13414-022-02525-z

**Published:** 2022-06-14

**Authors:** Stephanie C. Goodhew, Mark Edwards

**Affiliations:** Research School of Psychology, The Australian National University, Canberra, 2601 Australia

**Keywords:** Emotion, Attention, Anxiety, Emotion-induced blindness, Emotion attentional blink, Attentional bias

## Abstract

Scientists have long been interested in understanding the influence of emotionally salient stimuli on attention and perception. One experimental paradigm that has shown great promise in demonstrating the effect of such stimuli is emotion-induced blindness. That is, when emotionally salient stimuli are presented in a rapid stream of stimuli, they produce impairments in the perception of task-relevant stimuli, even though they themselves are task irrelevant. This is known as emotion-induced blindness, and it is a profound and robust form of attentional bias. Here, we review the literature on emotion-induced blindness, such as identifying the types of stimuli that elicit it, and its temporal dynamics. We discuss the role of dimensional versus categorical approaches to emotion in relation to emotion-induced blindness. We also synthesize the work examining whether certain individuals, such as those high in anxiety versus psychopathy, succumb to emotion-induced blindness to different extents, and we discuss whether the deficit can be reduced or even abolished. We review the theoretical models that have been proposed to explain the phenomenon. Finally, we identify exciting questions for future research, and elucidate useful frameworks to guide future investigations.

The physical world is complex and dynamic. Humans typically cannot process all of the available information at once, and therefore *attention* is a crucial psychological mechanism that triages particular stimuli for preferential processing at the expense of others. The question of what criterion or criteria are used in this triaging has long fascinated cognitive psychologists and cognitive neuroscientists (e.g., Fiebelkorn & Kastner, 2020; James, 1952). While attention influences the processing of information from all sensory inputs, vision is typically humans’ dominant sensory modality, and therefore the bulk of the scientific work has focused on selection in this domain—namely, *visual attention*.

Visual attention is affected by many factors, including the ease with which a stimulus can be differentiated from other concurrent stimuli (Treisman & Gelade, 1980), the relative similarity of task-irrelevant stimuli to the target of our goals (Becker, 2010; Becker et al., 2017), and whether a stimulus signals a behaviorally urgent threat, such as looming motion (von Muhlenen & Lleras, 2007). Emotions are an intrinsic part of the human experience, and it is perhaps for this reason that the power of the *emotional salience* of stimuli over visual attention has attracted much research interest. *Emotional salience* is a broad term that encompasses stimuli that evoke (e.g., gruesome scenes) or allude to (e.g., an image of a person showing a fearful facial expression) emotional experiences.^1^ While with prolonged exposure emotionally salient stimuli can influence a person’s emotional state, emotions have their own documented effects on attention (e.g., Fredrickson & Branigan, 2005; Gable & Harmon-Jones, 2008; Storbeck et al., 2018), which are dissociable from the effects that emotional salient *stimuli* have on attention on much shorter timescales (Goodhew & Edwards, 2022a). The interplay between emotional salience and visual attention has been the focus of decades of research. The extent to which emotionally salient stimuli have “special” attentional currency, such that they are capable of commandeering attentional systems for their prioritized processing even when they are irrelevant to an experimenter-prescribed task in the laboratory, has been of particular interest.^2^ This question is important because it provides insight into whether the attentional influence of such stimuli is sufficiently powerful or immutable that they can override any goals created by experimental instructions. In the early days following the advent of functional magnetic resonance imaging (fMRI), it was debated whether the human brain (especially the deep subcortical structure implicated in emotion, the amygdala) registered task-irrelevant emotionally salient stimuli. Initially, it was claimed that fearful (versus neutral) faces activated the amygdala irrespective of whether visual attention was applied to them, because faces in both task-relevant and task-irrelevant locations produced this activation (Vuilleumier et al., 2001). In contrast, subsequent studies indicated that a high perceptual load task moderated both fMRI-gauged amygdala responses to emotionally salient faces (Pessoa et al., 2002) as well as event-related potentials (ERPs) elicited by emotionally salient stimuli (Holmes et al., 2003). This fascination with the interaction between emotion and attention has resonated throughout the psychological and neuroscientific literatures.

One paradigm that has been used extensively to study the influence of task-irrelevant emotionally salient stimuli on attentional allocation is the *dot-probe*. In the dot-probe task, two visual stimuli are displayed concurrently (e.g., one above and one below central fixation) for a brief period of time (e.g., 500 ms), one of which is emotionally salient (e.g., a fearful face) and one of which is not (e.g., a neutral face). These images disappear, and then a probe (or target) that participants are instructed to respond to appears. This could be a dot that appears, which participants are required to detect or localize (hence, the name *dot-probe*), or it could be a letter which participants are required to identify (e.g., E versus F). The key metric is whether responses are faster (and/or more accurate) when the probe appears in the location that the emotionally salient stimulus previously occupied, compared with responses to the probe when it appears in the location that the neutral stimulus previously occupied (which is equally likely to be the upper or lower location). If they are, then this is considered to reflect an *attentional bias* (Bar-Haim et al., 2007; MacLeod et al., 1986; Mogg et al., 2004b). An influential meta-analysis indicated that this attentional bias effect is only observed in those with high levels of trait anxiety (Bar-Haim et al., 2007). However, more recent work has suggested that it may not even be reliable in this population (Kruijt et al., 2019).

The dot-probe has proved a useful tool for studying attentional processes with emotionally salient stimuli and how different individuals are more or less affected by such stimuli. However, a critique that can be levelled at the dot-probe is that it may not rigorously gauge the influence of truly *task-irrelevant* stimuli. This is important, because testing the effect of emotionally salient stimuli on attention when they are truly task-irrelevant is informative regarding their ability to command priority in the attentional system. In the dot-probe, during the time in which the images are displayed, participants have no competing task. Since the probe is equally likely to appear behind the emotionally salient stimulus as it is the neutral stimulus, this means that there is no cost to task performance for engaging with the emotionally salient stimulus (Basanovic & MacLeod, 2017; Brown et al., 2018). In other words, attending to the emotionally salient stimuli is inconsequential to task performance. This means that any attentional bias observed may not really reflect the capture of attention but could gauge more strategic or volitional decisions to allocate attention to the emotionally salient stimulus. Indeed, recent research has implicated “top-down” attentional processes in attentional bias inferred from the dot-probe (e.g., Delchau et al., 2020; Vogt et al., 2013). This raises the question of whether there is another paradigm for measuring attentional bias where allocating attention to the emotionally salient stimulus is demonstrably detrimental to task performance. While visual search is a paradigm that can satisfy this criterion, using this paradigm to assess attentional biases has its own issues, such as response efficiency being affected by low-level visual factors unrelated to emotional salience (Savage et al., 2013).

Furthermore, the dot-probe typically relies on response time as the dependent variable. However, there is evidence that while some forms of attention (e.g., nonpredictive spatial cues) influence only response efficiency (i.e., processing time and hence response time), other forms of attention (e.g., predictive spatial cues) can also influence perception (and therefore *accuracy* on a perceptual task; Prinzmetal et al., 2005; Prinzmetal et al., 2009). Accuracy can be considered a higher benchmark—indicating an actual change in how or whether a stimulus is ultimately perceived. For this reason, if emotionally salient stimuli can impact accuracy, then it would be informative to have an accuracy-based metric of attentional bias so as to better understand the influence of such stimuli on this important outcome measure, and for testing the role of individual and contextual factors in moderating these effects. While the dot-probe has been adapted to accuracy metrics (Van Damme et al., 2008), there is an effect arising from a different paradigm that demonstrates the influence of truly task-irrelevant emotionally salient on visual perception, as gauged by accuracy. It is called *emotion-induced blindness*.

A standard emotion-induced blindness paradigm entails a rapid serial visual presentation (RSVP) stream, where a series of visual images are presented briefly (e.g., 100 ms per item) and rapidly in a single location, typically the center of the screen. With the exception of the critical distractor, the images depict landscapes and streetscapes. Participants’ task is to identify the orientation of one target image, which is rotated 90° to the left or right. Critically, prior to this target image, a distractor image is presented, which is either emotionally salient or emotionally neutral. The distractor is not relevant to participants’ task (to identify the orientation of the target); however, emotionally salient distractors that appear near in time to the target produce a robust impairment in target identification accuracy relative to neutral distractors presented at the same point in the stream, and this is called emotion-induced blindness (Most et al., 2005). The time elapsed between the distractor and the target is quantified in terms of *lag* (e.g., Lag 1 = target is the item immediately following the distractor in the stream, or target 100 ms after distractor if 100 ms per item in the stream). Emotion-induced blindness is present at short lags (e.g., Lag 2), and diminishes until it is eliminated at later lags (e.g., Lag 8). Emotion-induced blindness is quantified as the difference in accuracy at a short lag (often Lag 2) between the trials with an emotionally salient distractor stimulus versus trials with a neutral distractor (Most et al., 2005, 2007).

The neutral distractor images are usually somewhat different from the filler images, and therefore there can be a slight decrement in target identification performance following the neutral distractors at short lags too, relative to a distractor-absent baseline conditions (Kennedy & Most, 2015a; Le Pelley et al., 2019). This means that the physical similarity between distractors and filler items plays a role in target perception in an RSVP stream, such that distractors that are discrepant from other items in the stream may adversely impact target perception (see also Asplund et al., 2010; Hoffman et al., 2020). However, emotion-induced blindness is typically referenced relative to this neutral baseline. That is, it is gauged as the difference in accuracy between the emotionally salient and emotionally neutral conditions at a given lag. This is useful because typically both emotionally salient and neutral distractors differ from the filler items, and so this comparison should control for this general stream-dissimilarity induced salience effect, selectively revealing the detriment specifically due to the emotional salience of the distractor.^3^

Readers familiar with the attentional blink (AB) may notice some similarities between the attentional blink and emotion-induced blindness. Both arise from an RSVP technique and can be considered gauges of temporal attention (i.e., how attention is allocated across time rather than space; Dux & Marois, 2009; Onie & Most, 2017). That is, attention is not monolithic, but instead consists of both spatial and temporal components, and emotion-induced blindness and the attentional blink in their standard forms both gauge temporal attention, in contrast the dot-probe, which gauges spatial attention (Onie & Most, 2017). While they both reflect temporal attention, the key methodological difference between emotion-induced blindness and the attentional blink is that in the attentional blink participants are searching for two targets, whereas in emotion-induced blindness, participants are searching for only one.^4^ As a consequence, the key conceptual difference is that emotion-induced blindness gauges the influence of a truly task-irrelevant distractor on target perception, in contrast to the attentional blink which gauges the influence of the first task-relevant target on perception of a second task-relevant target. However, despite these differences, as discussed later, there is evidence for shared mechanisms between the attentional blink and emotion-induced blindness.

Another difference between the dot-probe and emotion-induced blindness is that while individual studies have claimed attentional biases indexed from the dot-probe occur in unselected samples in some circumstances (e.g., Koster et al., 2004), meta-analytic evidence suggests that they are selective to individuals with high level of trait anxiety (Bar-Haim et al., 2007). However, more recently, the robustness of dot-probe derived attentional biases even in this population has been called into question (Kruijt et al., 2019). In contrast, emotion-induced blindness appears to occur robustly in unselected samples, making it a preferable candidate as measure of how humans generally engage with emotionally salient material.

The sections in this review will conform to the following structure: This section introduces emotion-induced blindness and contextualizes it in the broader literature on emotion-attention interactions. Following this, we will discuss some of the classic findings in emotion-induced blindness—namely, both the naturalistic categories of stimuli for which emotion-induced blindness is observed, and how learnt associations with otherwise-neutral stimuli can also induce emotion-induced blindness, and the important implications of these findings. Subsequently, we explain the findings regarding the temporal dynamics of emotion-induced blindness. Next, we discuss the core dimensions of emotion, valence and arousal, and their role in emotion-induced blindness, and also properties beyond valence and arousal that may be implicated in emotion-induced blindness. We then discuss the role of individual characteristics in the effect, and whether any contextual factors can reduce or eliminate the deficit. Following this, we will discuss theoretical models of selective attentional mechanisms that have been applied to explaining emotion-induced blindness. Finally, we highlight the most pressing and theoretically substantive research questions going forward that emotion-induced blindness can contribute to answering.

## Emotion-induced blindness and the types of stimuli that elicit it

In this section, we will introduce the paradigm that has been used to reveal emotion-induced blindness, and the some of the foundational studies that have demonstrated and explored this effect. We will also cover the diverse array of stimuli that elicit emotion-induced blindness. In particular, we will begin by considering how naturalistic images, both negative and positive valence, elicit emotion-induced blindness, and so too can emotionally salient words, but these appear less potent in their effect. Following this, we will consider the role of stimuli whose emotional salience is created in the laboratory via learned associations with punishment and reward. Finally, we also discuss the preliminary work using emotionally expressive faces to elicit emotion-induced blindness and highlight the need for future work with these stimuli.

In one of the foundational studies, Most and colleagues documented an effect that they named emotion-induced blindness, and drew the parallel with *rubbernecking*, the real-world phenomenon in which highway drivers often slow down to view accidents on the side of the road (Most et al., 2005). Emotion-induced blindness can be thought of as a laboratory attentional analogue of rubbernecking, in that people are drawn toward engaging with very negative emotionally salient material, even when it is unhelpful to the task at hand.

**Fig. 1:**
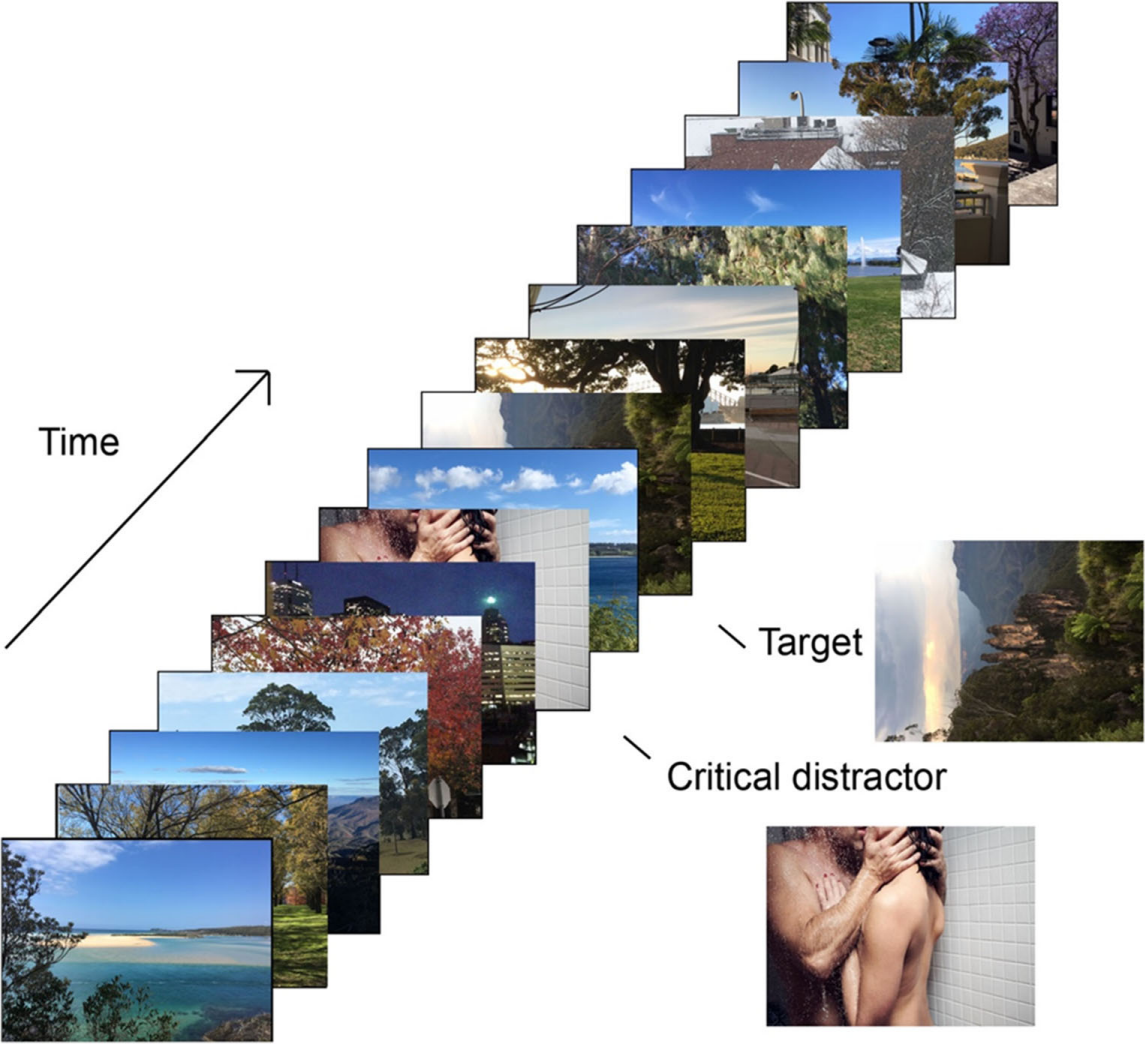
An illustrative example of an emotion-induced blindness experimental trial. *Note.* Each image is presented briefly (e.g., 100 ms). Participants’ task is to identify whether the target is oriented to the left or the right, and their accuracy is measured. The key manipulation is that the critical distractor is either emotionally salient (negative or positive) or emotionally neutral, and the time (lag) between the distractor and target is varied. This example uses an image similar to those typically used as a positive critical distractor, although those used in research often depict more explicit nudity and sexual acts. The critical distractor image was obtained from unsplash.com; all other images were photographs taken by the first author.

**Fig. 2:**
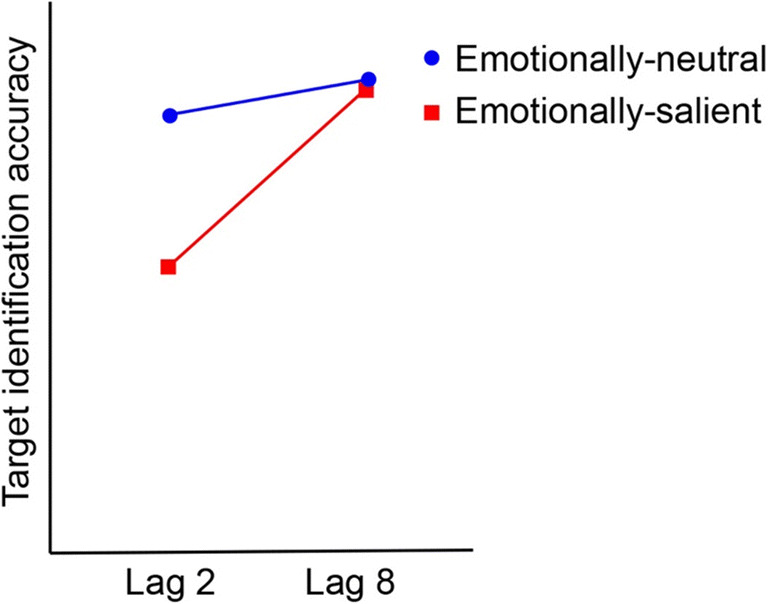
An illustrative example of results from an emotion-induced blindness procedure. *Note.* There is typically an interaction between lag and type of distractor, such that there is a pronounced decrement in target identification accuracy following the critical distractor relative to that following the neutral distractor at an early lag (e.g., Lag 2), which has dissipated by a later lag (e.g., Lag 8). Emotion-induced blindness is typically quantified as the difference in accuracy between the emotionally salient and emotionally neutral distractor conditions at Lag 2.

Most et al. (2005) used a rapid serial visual presentation stream containing 17 color images and varied the lag between the critical distractor and the to-be-identified target in the stream. Each image was 15-cm wide × 1-cm^5^ high on the computer screen and was presented for 100-ms, and the target appeared either 200 ms or 800 ms after the distractor (i.e., Lag 2 or Lag 8). The target was rotated 90° to the left or right of vertical, and participants’ task was to identify its orientation. The distractor images were either negative valence^6^ (e.g., pictures of graphic violence) or neutral. The key finding was an interaction between the factors of lag and distractor valence, such that target orientation identification accuracy was differentially reduced following the negative distractor at Lag 2 relative to accuracy following the neutral distractor, a difference that was abolished at Lag 8. Emotion-induced blindness is typically operationalized as the difference in accuracy following an emotionally salient versus an emotionally neutral distractor at a short lag, such as Lag 2 (Most et al., 2005).^7^ This effect has also been repeatedly replicated in later studies (e.g., Kennedy & Most, 2012, 2015b; Kennedy et al., 2018a, b; Most et al., 2006; Most & Jungé, 2008; Onie & Most, 2017; Proud et al., 2020; Zhao & Most, 2019). For an example of an emotion-induced blindness experimental procedure and indicative results, see Figs. 1 and 2.

Most et al. (2005) also used a control distractor stimulus that consisted of a scrambled version of the negative pictures as a control for the low-level perceptual aspects (e.g., color) of the images. These images did not produce an impairment in target perception in the way that the intact negative images did. Subsequent emotion-induced blindness work has not used the scrambled-image distractor as a control. While these scrambled images would contain overlapping basic perceptual information with the intact negative images, there are also important differences, such as in local contour information. They therefore do not provide the ideal control stimulus for low-level perceptual factors, however, subsequent work using conditioning to create emotional salience for different stimuli and employing these as distractors have decisively refuted the notion that emotion-induced blindness is merely a product of low-level perceptual factors.

That is, a complementary approach to operationalizing the emotional salience of stimuli in emotion-induced blindness is to take otherwise neutral stimuli and *create* the emotional salience of the stimuli in laboratory. This approach has been used successfully in other domains—for example, showing spatial attentional effects resulting from stimuli associated with punishment (Anderson & Britton, 2020; Schmidt et al., 2015; Wentura et al., 2014) or reward (Anderson et al., 2011; Failing et al., 2015; Le Pelley et al., 2022). This association is created via a learning process, in which the participant learns that a particular type of stimulus (e.g., a green disc) predicts an aversive outcome (e.g., mild electric shock) or a positive one (e.g., reward), whereas a similar stimulus (e.g., a red disc) does not. Also, typically it is counterbalanced which stimuli are associated with different outcomes across participants, thereby eliminating any stimulus-specific explanation for effects when averaging across participants. For example, for half of participants, green discs images are imbued with emotional salience via their association with outcomes and red discs are the control stimulus, whereas for the other half of participants, this mapping is reversed. This setup allows for excellent experimental control over the low-level physical properties of the stimuli. Then, if there is a systematic difference in performance resulting from the stimuli with emotional salience, this cannot be attributed to low-level physical properties alone. As discussed below, this approach has been taken to induce both positive and negative associations with stimuli that would otherwise be devoid of emotional salience, and such stimuli induce a standard pattern of emotion-induced blindness. This is compelling evidence that emotion-induced blindness is the product of *emotional salience* of the critical distractor, rather than masking or interference due to the low-level visual properties of stimuli that naturally or intrinsically have emotional salience.

The first of the studies on emotion-induced blindness to adopt the learnt-association approach to manipulating emotional salience used stimuli that ordinarily have neutral emotional salience (i.e., pictures of birds or cars), and via a classical conditioning procedure paired these with an aversive stimulus (i.e., a blast of white noise; Smith et al., 2006). Whether the birds or the cars were associated with the aversive stimulus (i.e., whether the birds or cars were CS+) was counterbalanced across participants. Subsequently, the CS+ produced an impairment in target perception when it appeared close in time prior to the target in a rapid serial visual presentation stream (i.e., Lag 2), an effect which disappeared at longer distractor-target intervals (i.e., Lag 8). In other words, these stimuli produced emotion-induced blindness (Smith et al., 2006). These authors also refuted the alternative explanation that any visual stimulus associated with a sound would produce emotion-induced blindness via a control experiment in which the stimuli were associated with a non-aversive sound (i.e., ocean wave sound). Such stimuli did not produce emotion-induced blindness, thereby demonstrating that it is selectively stimuli that have learned *emotionally salient* associations that produce emotion-induced blindness (Smith et al., 2006). In a similar vein, it has been shown that participants who watched a trauma-related film experienced emotion-induced blindness for images that were otherwise-neutral but served as reminders of the traumatic film (Verwoerd et al., 2010). It has also been demonstrated stimuli that signal the availability of punishment can elicit emotion-induced blindness (Le Pelley et al., 2019). Thus, both naturalistic negative stimuli and those with learned associations with negative outcomes can induce emotion-induced blindness.

Following on from the work with negative-valence distractor images, Most et al. (2007) tested whether emotion-induced blindness occurs for high-arousal positive images. These authors noted that while previous studies had suggested that positive images may have less impact on attention than negative ones, in such studies, the positive images used were often only mildly positive with intermediate- or low-arousal levels, whereas the negative images were intensely negative with high-arousal levels. This means that any difference in their effect on attention could be attributed to their intensity (i.e., their degree of valence), rather than to which valence polarity (i.e., positive or negative) they belonged. Most et al. (2007) solved this issue by selecting emotionally salient distractor images that were rated to be highly positive and arousing. The participants were male, and the images that they rated as most highly positive and arousing were images of female nudes, while control-condition images included clothed females and clothed males. The other aspects of the design were similar to that in the original negative-image study (Most et al., 2005). A strong pattern of emotion-induced blindness emerged once again: Participants’ target orientation identification accuracy was substantially worse following the presentation of a nude female distractor close in time prior to the target (i.e., Lag 2) compared with accuracy following either the clothed female or clothed male distractor, and this difference was abolished with a greater interval of time between the distractor and the target (i.e., Lag 8; Most et al., 2007). In other words, emotion-induced blindness also occurs with positive-valence emotionally salient distractors. This form of emotion-induced blindness was also invariant to the offer of financial reward for accurate target identification responses (Most et al., 2007).

While the bulk of the literature on emotion-induced blindness has used photographic images of naturalistic images, the effect of word-stimuli has also been investigated. It has been found that emotion-induced blindness also occurs following sexual/taboo critical distractors (e.g., *orgasm*), but not following generic positive (e.g., *beauty*) or negative valence (e.g., *broken*) words (Arnell et al., 2007). This absence of emotion-induced blindness following generally negative or positive valence words is likely because words are less emotionally potent than images, and thus the effect is limited to only the most evocative words.

The conditioning approach to imbuing otherwise-neutral stimuli with emotional salience has also been adopted to create positive valence distractors. In particular, value-modulated attentional capture that was first documented in the spatial domain (Le Pelley et al., 2015; Pearson et al., 2015) has been applied in the temporal domain. That is, Le Pelley et al. (2017) discovered that stimuli that signaled the availability of reward (but were not themselves targets) could serve as distractors to produce emotion-induced blindness. Such effects persisted during an extinction phase when rewards were no longer available and occurred even when the stimuli were only probabilistic signals of rewards and deterministic information regarding the availability of reward was provided at the beginning of the trials (Le Pelley et al., 2017). This means that learned positive associations can produce emotion-induced blindness (see also Le Pelley et al., 2019).

Further evidence that stimuli with learned positive-valence emotional-salience can produce emotion-induced blindness comes from a study by Gutiérrez-Cobo et al. (2019) in which they varied two key factors: (a) whether the critical distractor was an angry or a neutral face, and (b) whether each face was associated with reward (i.e., points, and then ultimately money). In Experiment 1, the emotional expression of the face determined whether the face was associated with reward or not, and in Experiments 2 and 3 it was the gender of the face that signaled this. Across all three experiments, both of these factors had an effect on target perception: target identification accuracy was lower following angry versus neutral faces, and the same was true following reward-associated versus non-reward-associated stimuli.

Humans are social animals, and the emotional expression of faces can therefore hold emotional significance (e.g., Jack & Schyns, 2015). Given this, emotion-induced blindness has been applied to investigating whether images of faces exert a detrimental effect on target perception, even though they are not task relevant. The small number of studies that have investigated this question have led to mixed results. Gutiérrez-Cobo et al. (2019) found a consistent main effect whereby target identification accuracy was lower following the angry face versus the neutral face distractor, indicative of emotion-induced blindness elicited by the angry face (Gutiérrez-Cobo et al., 2019). In contrast, an earlier study that was couched in terms of attentional blink but actually resemble emotion-induced blindness parameters did not find this effect when the emotion of the face was not task relevant. That is, Stein et al. (2009) examined the influence of using fearful versus neutral expression faces as T1 in an attentional blink (i.e., rapid serial visual presentation with two prescribed targets). These authors varied how task-relevant the faces were across three experiments. The targets were scenes, and filler items were scrambled images. These authors found that when participants made an emotion judgement (i.e., fearful versus neutral) on the T1 images, the fearful faces produced a greater blink than did neutral faces. In contrast, this effect was lost when the faces were entirely task-irrelevant and participants did not respond to them (i.e., akin to emotion-induced blindness; Stein et al., 2009). These results would appear to contrast with those of Gutiérrez-Cobo et al. (2019), in which task-irrelevant angry faces did elicit emotion-induced blindness. It could be that angry faces elicit emotion-induced blindness whereas fearful ones do not. Alternatively, the different face databases the images were drawn from in the two studies (i.e., Ekman versus Nimstim) could be responsible. Alternatively, another explanation for the contrasting results between these two studies is that as pointed out by de Jong et al. (2014), Stein et al. (2009) used a fixed-temporal position in the stream for the target (i.e., target always tenth item), which other evidence indicates participants quickly learn and this can mitigate temporal-attentional impairments (Tang et al., 2014). This may have made the paradigm less sensitive to the influence of other variables, such as the emotional salience of the critical distractor. However, another study found that angry and happy expression faces *facilitated* target perception at Lag 2 (de Jong et al., 2014). Altogether, these mixed findings suggest that more work needs to be done to understand the influence of emotionally expressive faces on emotion-induced blindness.

To summarize, emotion-induced blindness refers to the decrement in target perception that occurs when the target appears close in time after an emotionally salient distractor. Emotion-induced blindness occurs following naturalistic images, including both negative (e.g., mutilated bodies) and positive (e.g., erotic) images, and stimuli with learned associations, including both those associated with an aversive outcome and those associated with reward. The finding that stimuli whose emotional salience is learned within the context of a laboratory experiment compellingly demonstrates that it is emotional salience, rather than some low-level visual property that systematically varies as a function of emotional salience that is responsible for the observed effects. The role of faces with different emotional expressions in emotion-induced blindness is currently not well understood, as the few studies that have used faces have obtained conflicting results.

## The temporal dynamics of emotion-induced blindness

As illustrated in the previous section, the original and most commonly employed time-based manipulation in emotion-induced blindness is to compare the effect of different critical distractors at Lag 2 (i.e., target second item after critical distractor, or 200 ms after the onset of the distractor if 100 ms/image) versus Lag 8 (i.e., target eighth item after critical distractor, or 800 ms after the onset of the distractor). Emotion-induced blindness—that is, the difference in target identification accuracy between when an emotionally salient and emotionally neutral distractor is used in the rapid serial visual presentation stream—is typically restricted to Lag 2 and eliminated by Lag 8. This shows that the distractor affects the perception of *subsequent* stimuli. However, other work has also examined more fully the temporal dynamics of emotion-induced blindness.

In particular, across two experiments, Most and Jungé (2008) examined the temporal dynamics of emotion-induced blindness induced by negative versus neutral distractors at varying intervals between the distractor and target. In Experiment 1, they compared performance when targets appeared *before* the critical distractor (lag minus 1) with performance when the target appeared at Lag 2. Emotion-induced blindness was observed for both lags. Indeed, individuals’ performance correlated between these two lags, which is consistent with common mechanisms underlying performance in both conditions. In Experiment 2, there was no emotion-induced blindness when the target appeared in position lag minus 2, whereas it was present at Lag 1. In this experiment, performance at these two different lags did *not* correlate, which is consistent with them reflecting distinct mechanisms. Altogether, this indicates that the distractor can also affect target perception retrospectively, if the target and distractor appear in a similar temporal window (Most & Jungé, 2008).

Emotion-induced blindness has been found to occur irrespective of whether participants are instructed to wait until the end of the stream to respond or whether they respond immediately upon seeing the target (Kennedy & Most, 2012). This has led to the conclusion that emotion-induced blindness is predominately an attentional-perceptual effect, rather than a memory-based one (Kennedy & Most, 2012). Finally, recent work has also shown that context (i.e., threat of shock) can prolong the temporal dynamics of emotion-induced blindness (Haddara et al., 2019).

In summary, it appears that emotion-induced blindness is not limited to Lag 2, but also occurs at Lag 1, and even lag minus 1 (when the target precedes the distractor) but is not present at lag minus 2. This tells us that the distractor can exert an influence on perception of a prior target, but only over a tightly limited timeframe. An explanation for these effects is offered in the section discussing theoretical models of emotion-induced blindness.

## The building blocks of emotion and their role in emotion-induced blindness

What are the fundamental building blocks of emotion? Psychological scientific approaches to answering this question have typically subscribed to one of two key schools of thought: (a) that there are a handful of discrete and basic human emotions that are universally experienced and recognized, such as fear, anger, and happiness (Cordaro et al., 2018; Ekman, 1992; Ekman & Friesen, 1971; Ekman et al., 1983; Izard, 1992), versus (b) that emotion can be distilled down to values along key dimensions—most commonly valence (bipolar; unpleasant → pleasant, with neutral in between) and arousal (unipolar; low arousal → high arousal; Bliss-Moreau et al., 2020; Colibazzi et al., 2010; Cuthbert et al., 2000; Fredrickson & Branigan, 2005; Lang et al., 1998; Lang et al., 1993; Olofsson et al., 2008; Russell, 1980). There is evidence that both of these approaches have merit (Grootswagers et al., 2017), and it is not our goal here to adjudicate between them, but instead to review and evaluate how these different approaches have been applied to understanding the attentional biases reflected in emotion-induced blindness, and ultimately make recommendations for future research.

The majority of the work on emotion-induced blindness has focused on the dimensions of valence and arousal. That is, stimuli which are polarized with respect to valence (i.e., highly positive or highly negative) and concurrently high in terms of arousal elicit emotion-induced blindness (e.g., Most et al., 2005, 2007). Valence and arousal typically go hand in hand, such that stimuli (or emotions) that have polarized valences also tend to be highly arousing, while those with low-arousal levels also tend to have more neutral or middling valences. This means that when a series of stimuli are plotted as points in a scatterplot where the valence value of the emotion they induce is on the *x*-axis and arousal value on the *y*-axis, a U-shaped scatterplot emerges. While they are not completely independent, valence and arousal have been found to be dissociable in terms of the physiological responses they produce (Colibazzi et al., 2010), as well as their impact on other cognitive processes like memory (Kensinger & Corkin, 2004).

Since both highly positive and highly negative stimuli can produce emotion-induced blindness, it has typically been concluded that it is the arousal value rather than valence value of stimuli that determines their level of emotional interference (Most et al., 2007). However, while emotion-induced blindness is not limited to a particular valence of critical distractor, it could be that valence is important in another way, such as that stimuli with extreme deflections from neutral in either direction on valence produce emotion-induced blindness. It is only relatively recently that both valence and arousal have been systematically varied and their unique effects on emotion-induced blindness assessed. Across two experiments, Singh and Sunny (2017) experimentally varied valence versus arousal level of the emotionally salient distractor while holding the other dimension constant and examined the impact of this on the magnitude of emotion-induced blindness. They found that arousal, rather than valence, was the critical determinant of emotion-induced blindness magnitude. However, more recent work has suggested that both valence and arousal uniquely contribute to emotion-induced blindness (Onie & Most, 2021).

A categorical approach to understanding emotion features far less in the literature on emotion-induced blindness. However, Perone et al. (2020) compared disgust-eliciting, fear-eliciting, and emotionally neutral stimuli as distractors in emotion-induced blindness. Both when participants were judging whether the target was present or absent (i.e., target detection) and when judging whether the target was left or right oriented (i.e., target identification), disgust stimuli led to a greater detriment in performance than fear stimuli, which both produced worse performance than neutral stimuli at Lag 2. This was so even when controlling for the valence and arousal values of the stimuli. The authors concluded that humans may spend longer analyzing pathogens, and they call this effect a manifestation of the “behavioral immune system” (Perone et al., 2020).

In summary, from a dimensional perspective, there is evidence that both valence and arousal contribute to the magnitude of emotion-induced blindness. To date valence and arousal have only been pitted against one another as predictors of emotion-induced blindness following naturalistic images, and therefore it remains to be tested these findings generalize to emotion-induced blindness elicited by stimuli that acquire their emotional salience from learned associations. From a categorical perspective, it appears that disgust may preferentially boost the magnitude of emotion-induced blindness over other negative-valence emotions like fear. However, applying this approach is relatively novel, and more work needs to be done here. We provide some suggestions in the section on future research directions later in this piece.

## Are some individuals more or less susceptible to emotion-induced blindness?

From reviewing the literature above, it is clear that emotion-induced blindness is a robust effect. From our own experience, it is *not* one of those fleeting or elusive laboratory phenomena—it is so strong and robust that you can subjectively *experience* it from a single example trial, and see it clearly manifest consistently in individual participants’ data. There has been considerable research interest in whether the magnitude of emotion-induced blindness relates to individual-difference variables, such as a person’s mood or ability to regulate attention. However, as we review below, in contrast to the robustness of emotion-induced blindness itself, its relationship with individual-difference variables is less consistent.

In the original Most et al. (2005) study, the authors examined the *harm avoidance* component of the Tridimensional Personality Questionnaire (Cloninger et al., 1991). High harm avoidance includes being anxious, tense, risk-avoidant, and slower to recover from stress, whereas low harm avoidance includes being carefree and confident, a tendency to take risks, and a quicker recovery from stress. Most et al. (2005) found no main effect of harm avoidance of emotion-induced blindness. However, when the authors varied target specificity (i.e., instructed participants to search for building [specific] versus building or landscape [nonspecific], while this also had no overall impact on emotion-induced blindness, it did interact with harm avoidance in its impact. That is, individuals with high levels of harm avoidance had reduced emotion-induced blindness in the target-specific condition only (Most et al., 2005). However, a subsequent study did not replicate this moderation effect (Most et al., 2006).

In a seminal study, Onie and Most (2017) emphasized distinction between spatial and temporal attention. Therefore, in Experiment 1, they compared performance on the dot-probe versus emotion-induced blindness for negative (versus neutral) material to gauge spatial versus temporal attentional biases to negative material. They found no relationship between emotion-induced blindness and dot-probe attentional bias metrics. Moreover, each of these measures predicted unique variance in self-reported negative affect in everyday life (Onie & Most, 2017), as measured by the Depression Anxiety and Stress Scale (DASS; Lovibond & Lovibond, 1995; Lovidbond & Lovibond, 1995).

In a similar vein, emotion-induced blindness has been found to be associated with issues in the termination*,* rather than initiation of worry (Berenbaum et al., 2018). That is, in this study, participants were prompted on multiple occasions each day to answer a variety of questions about their worrying. Worry initiation was operationalized as the onset of worrying about new topics, while continuing to worry about the same topics and the duration of worrying were attributed to difficulty with the termination of worry. Individual differences in the latter, but not the former, were associated with the magnitude of emotion-induced blindness (Berenbaum et al., 2018).

In another study, participants with high levels of trait anxiety (as measured by the State-Trait Anxiety Inventory [STAI]; Spielberger et al., 1983) showed greater emotion-induced blindness at early lags (Chen et al., 2020). However, there have been a number of failures of replicate such associations. For example, both Kennedy et al. (2020) and Perone et al. (2020) found no relationship between negative affect (DASS) and emotion-induced blindness. Similarly, Guilbert et al. (2020) found no relationship between anxiety as measured by the subscale of that name from the DASS and emotion-induced blindness. Another study found that patients with obsessive-compulsive disorder (OCD) showed a selective impairment relative to controls following erotic images, but not following fear or disgust distractors. However, performance was not related to trait anxiety, as measured via the STAI (Olatunji et al., 2011b).

The relationship between emotion-induced blindness and individual-differences in attentional control, as measured by the Attentional Control Scale (ACS), has also been the focus of several investigations. ACS measures the ability to focus attention and to efficiently switch tasks in everyday life and is also associated with depression and anxiety (Derryberry & Reed, 2002; Judah et al., 2014). One study found that with a specific attentional set, individuals high in attentional control were able to improve performance selectively on the neutral, but not on the negative, trials. This resulted in a larger difference between neutral and negative distractor trials, thereby resulting in a larger emotion-induced blindness magnitude for these individuals high in attentional control in this specific attentional set condition (Most et al., 2006). A similar advantage for targets at short lags for those high in attentional control has been observed elsewhere, but there the advantage was particularly clear if the distractor was emotional rather than neutral (Peers & Lawrence, 2009). Markedly poorer performance for individuals diagnosed with Generalized Anxiety Disorder following neutral distractors has also been found to be mediated by individual differences in attentional control (Olatunji et al., 2011a). Furthermore, across three experiments, Kennedy et al. (2018a) found inconsistent relationships between depression (as measured by the Beck Depression Inventory [Beck et al., 1961], with the suicide question removed), attentional control (ACS), harm avoidance, and the performance benefit due to warnings (i.e., reduction in emotion-induced blindness when participants are provided with foreknowledge about the type of distractor on a given trial, a manipulation discussed in more detail in the next section). Altogether, on the whole, there appears to possibly be some relationship between attentional control and performance in single-target rapid serial visual presentation tasks, but there is inconsistency regarding the nature of this relationship.

Some studies have found that the alignment between the types of stimuli that produce impairment and the nature of the individual-difference variable of interest is important in determining emotion-induced blindness magnitude. Olatunji et al. (2013) found impaired target perception following combat-related distractors in veterans with posttraumatic stress disorder (PTSD) relative to either veterans without PTSD or healthy controls. This impairment was selective to combat-related distractors, as the veterans with PTSD were unimpaired following disgust, positive, or neutral distractors.

In a similar vein, Borton et al. (2017) compared individuals with defensive self-esteem (i.e., high-explicit and low-implicit self-esteem) with those with secure self-esteem (i.e., high-explicit and high-implicit self-esteem). They found that those with defensive self-esteem exhibited greater emotion-induced blindness following a face signaling social rejection (i.e., disgust expression), compared with both other negative emotionally arousing images (i.e., violence and medical trauma), and compared with an accepting (i.e., smiling) face, relative to individuals with secure self-esteem. This was so at Lag 4, but not at Lag 2 or Lag 6 (Borton et al., 2017).

Reduced distraction following negative distractors has been found for individuals who play violent videogames (Jin et al., 2018) and for individuals from a community sample scoring high on aspects of psychopathy (Kimonis et al., 2020). A similar pattern of reduced distraction from negative emotionally salient material is seen in older adults, although this presumably reflects a different a mechanism from that underlying those high in psychopathy or experienced violent videogame players. That is, Kennedy et al. (2020) examined emotion-induced blindness following negative and positive distractors in younger and older adults. Across a series of experiments, older adults did *not* experience emotion-induced blindness following negative distractors, despite these same stimuli being effective inducers for younger adults, whereas the older adults did experience emotion-induced blindness following positive distractors. This is consistent with the general tendency for a positivity bias to be present in older adults (Charles et al., 2003; Kennedy et al., 2020).

In summary, the magnitude of emotion-induced blindness has been found to relate to several individual-difference variables, such as negative affect and attentional control, but the literature is mixed, with a number of failures to replicate previously observed effects. In the section on future research directions, we discuss what we believe may be the root cause of these inconsistencies and explain how this can be addressed going forward.

## Can emotion-induced blindness be reduced?

Emotion-induced blindness reflects a perceptual interference from task-irrelevant material, and at least some of the time it is associated with experiences of negative affect in everyday life. Can this detrimental intrusion be reduced, or even abolished entirely? Here, we review some of the key findings on this issue, which highlights that emotion-induced blindness is a remarkably stubborn phenomenon.

Kennedy et al. (2018a) examined the extent to which forewarning about the nature of a distractor could engage *proactive attentional control* and reduce the target-identification impairment produced by emotional distractors. Proactive control reflects the sustained and anticipatory maintenance of goal-relevant information, in contrast with reactive control where it is transient stimulus-driven goal reactivation (Braver, 2012). To operationalize proactive attentional control, Kennedy et al. (2018a) provided participants with trial-by-trial information about the nature of the distractor and measured the impact on emotion-induced blindness compared with when participants were not given this information. They used both positive and negative (versus neutral) distractors. In Experiment 1, participants were given the instruction at the start of the trial to “Ignore gruesome” or “Ignore erotic” or “Unknown.” Foreknowledge of the distractor type improved target identification accuracy Lag 2 following both negative and erotic distractors, and at Lag 4 for targets following negative but not erotic distractors. In Experiment 2, the warning was simply “graphic” for both negative and erotic. This warning improved target identification performance following both distractor types at Lag 2, and improved performance following erotic but not negative distractors at Lag 4. However, as the authors highlight, in all cases, the benefit due to proactive control was modest, and emotion-induced blindness was *never* eliminated (Kennedy et al., 2018a). This indicates that while proactive attentional control deployed in response to warning about the type of distractor that was going to appear on that trial can modulate the magnitude of emotion-induced blindness, the presence of emotion-induced blindness remains robust.

In a similar vein, Zhao and Most (2019) assessed the impact of the proportion of trials on which the critical distractor appeared on emotion-induced blindness. This work was inspired by previous research showing that non-emotional effects such as the Stroop effect are modulated by the proportion of incongruent trials, such that the effect is reduced as the proportion of incongruent trials increases. Such effects can be attributed to the recruitment of proactive control (Cheesman & Merikle, 1986). Furthermore, in the domain of emotional salience and *spatial* attention, it appears that the frequency with which an emotional distractor appears modulates the degree of attentional capture by that distractor, such that capture reduces with increasing frequency (Grimshaw et al., 2018). Across four experiments, Zhao and Most (2019) manipulated the proportion of trials in a block for which a negative versus a neutral distractor appeared, such that it was either high or low. The results were clear: Emotion-induced blindness was present, and it was not moderated by the frequency of emotional distractor trials (Zhao & Most, 2019).

Another approach to attempt to reduce emotion-induced blindness is to enhance perception of the target, rather than diminish the effect of the distractor. In this vein, Guilbert et al. (2020) found that using familiar stimuli (e.g., picture of Sydney Opera House) as targets increased overall target perception but affected both valences (i.e., emotionally salient and emotionally neutral conditions) equivalently, such that target familiarity did not modulate the magnitude of emotion-induced blindness. This was so even though ratings confirmed that participants did consider the selected familiar stimuli as reliably more familiar (Guilbert et al., 2020).

In summary, varying the frequency of emotionally salient distractor trials appears to have no discernible effect on emotion-induced blindness at all. This stands in contrast to how this manipulation can eliminate the distracting effect of emotionally salient stimuli on spatial attention. Increasing familiarity of the targets also did not reduce emotion-induced blindness. Proactive control was one factor that was found to reduce emotion-induced blindness to some extent, but even this did not eliminate it. Altogether, emotion-induced blindness is strikingly robust in the face of the contextual variables tested to date.

## Why does emotion-induced blindness occur? Theoretical models of emotion-induced blindness

One classic model for understanding attentional bottlenecks in RSVPs streams is the two-stage model (Chun & Potter, 1995). According to this model, the briefly presented stimuli enter Stage 1 of processing which entails just initial detection of the stimuli and is a temporary and fragile level of representation. Multiple items from the RSVP stream can be represented at any one time in Stage 1, but here items are susceptible to decay and easily overwritten by subsequent stimuli that enter this stage. Stage 2 entails a more robust representation of stimuli, of the kind that supports explicit perception and report of the stimulus. However, the consolidation of items from Stage 1 into Stage 2 is sluggish and capacity limited, creating a bottleneck. This means that items can be lost from Stage 1 before they are consolidated into Stage 2. This account was originally proposed to explain why the attentional blink occurs: while the first target is being consolidated into Stage 2, a close-in-time second target is at Stage 1 awaiting consolidation during this time, making the second target vulnerable to being overwritten by subsequent stimuli (Chun & Potter, 1995). However, it can also explain why emotion-induced blindness occurs: the emotionally salient distractor commandeers consolidation into Stage 2 over the target, leaving it vulnerable to being overwritten by subsequent stimuli.

The two-stage model can also account for why a distractor that appears after the target can still produce emotion-induced blindness: Stage 1 representations persist briefly beyond the physical presentation time of the stimulus, meaning that emotion-induced blindness can occur whenever the distractor and target are both at Stage 1 needing consolidation into Stage 2, which can happen whenever they are in tight temporal proximity (Chun & Potter, 1995; Most & Jungé, 2008). Indeed, the two-stage model is capable of explaining similar effects in the attentional blink literature, such as that the second target can affect perception of the first if they appear in close temporal proximity (Chun & Potter, 1995). However, another model that favors spatially localized mechanisms over central attentional bottlneck mechanisms (Most & Wang, 2011) can also explain both the presence of standard emotion-induced blindness, as well as the ability of a subsequent distractor to affect target perception.

Most and Wang (2011) investigated the theoretical mechanism underlying emotion-induced blindness, and ultimately proposed the spatiotemporal competition account of emotion-induced blindness. In doing so, they drew upon existing evidence of neural competition for spatiotemporal dominance (Desimone & Duncan, 1995), which has been proposed to explain a diverse array of perceptual phenomena including masking and binocular rivalry (Keysers & Perrett, 2002). From this perspective, the brain only allows a single representation to “win” the competition for consciousness when two stimuli are presented close in time at the same spatial location, because in the real world, two objects cannot occupy the same spatial location at the same time. Keysers and Perrett (2002) highlight that representations persist beyond the physical presentation of a stimulus, meaning that there can be overlap in representations for sequentially presented stimuli. Emotion-induced blindness could therefore be the result of the emotionally salient distractor stimulus winning this spatiotemporal competition for consciousness when it appears close in time before or after the target.

Most and Wang (2011) sought to determine whether emotion-induced blindness can be explained by a “central” (i.e., nonspatially specific) deficit, in which case the effect should be spatial location invariant as would be predicted from an attentional bottleneck account, or whether it is a spatially localized effect, and therefore better explained by a spatially specific mechanism, such as competition for spatiotemporal dominance. To do this, they used a dual-stream methodology, in which two concurrent, spatially offset streams were presented, and the distractor and target could appear in either of them. They found that when the critical distractor appeared in the same stream as the target, emotion-induced blindness occurred. In contrast, when the distractor appeared in one stream and the target in the other, then emotion-induced blindness did not occur (Most & Wang, 2011). This suggests that emotion-induced blindness is spatially localized, which is consistent with the spatiotemporal dominance account (Most & Wang, 2011; Wang et al., 2012).

However, the notion of emotion-induced blindness intrinsically reflecting a spatially localized deficit has been challenged. Proud et al. (2020) argued that this evidence for spatial localization arises from a vigilance-avoidance mechanism in response to negative stimuli in anxious individuals, and that once this is accounted for, emotion-induced blindness can be explained by a central-level (i.e., non-spatially-specific) attentional bottleneck. Vigilance avoidance is a cognitive reaction to threat by individuals with high levels of anxiety, which manifests as initial enhanced visual engagement followed by rapid disengagement from and avoidance of threat (Blicher & Reinholdt-Dunne, 2019; Derakshan et al., 2007; Koster et al., 2006; Mogg & Bradley, 1998; Mogg et al., 2004a; Weinberg & Hajcak, 2011). For example, with the dot-probe paradigm, the pattern that has been attributed to vigilance avoidance is that where high-trait-anxious individuals show strong bias toward with threatening stimuli at 100 ms after exposure, but strong bias away by 500 ms (Koster et al., 2006). Within the context of dual-stream emotion-induced blindness, vigilance avoidance could make anxious participants move their attention to the opposite stream following the presentation of a threat distractor, thereby boosting target perception in that opposite stream, which in turn reduces emotion-induced blindness for that stream. At the same time, this would impair target perception in the stream that the distractor appeared in, hence giving the appearance of spatially localized emotion-induced blindness (Proud et al., 2020). That is, vigilance avoidance following the presentation of a negative distractor would produce the pattern of results observed in Most and Wang (2011).

How can we distinguish between these two theoretical accounts? Proud et al. (2020) reasoned that the spatiotemporal competition account predicts that the spatial localization of emotion-induced blindness should occur for *all* emotional stimuli (i.e., both positive and negative stimuli), and for *all* participants (irrespective of their level of anxiety). In contrast, according to the vigilance-avoidance account, vigilance-avoidance mechanism ought to be contingent on both (a) negative stimuli being used, the mechanism should not operate in response to positive stimuli if they are not perceived as threatening, and (b) this apparent spatial-localization effect for negative stimuli should only occur in individuals with high levels of anxiety, not those with low levels of anxiety. In other words, the vigilance-avoidance account predicts an interaction between stimulus valence, and individuals’ trait anxiety. This is exactly what Proud et al. (2020) found. That is, they found that emotion-induced blindness was *not* spatially localized following positive distractors irrespective of participants’ trait anxiety levels (as gauged via STAI), and it was not spatially localized following negative stimuli for participants with low levels of anxiety. Instead, the only spatially localized pattern of interference occurred for highly anxious individuals following negative distractors (Proud et al., 2020).

Proud et al. (2020) argued the apparent evidence for spatial-localization of emotion-induced blindness results from vigilance avoidance, rather than reflecting an intrinsic hallmark of emotion-induced blindness. These authors therefore argued that the two-stage model can still offer a viable account of emotion-induced blindness once vigilance-avoidance is accounted for. Notably, the spatial-localization model of emotion-induced blindness cannot explain why Proud et al. (2020) found observed emotion-induced blindness in both streams (i.e., including when the distractor and target were in different streams) following the positive stimuli for all participants, and observed it following the negative stimuli for participants with lower levels of anxiety.

Further, the spatially localized effect following negative distractors in dual-stream emotion-induced blindness has been found to occur at Lag 2 but not Lag 1 (Wang & Most, 2016). Since emotion-induced blindness itself is present at Lag 1 (Most & Jungé, 2008), the finding that the spatially localized effect does not emerge until Lag 2 is consistent with it arising from a different mechanism (such as vigilance avoidance) compared with emotion-induced blindness itself. More specifically, the finding that the spatially-localized effect emerges 200 ms after the negative distractor is consistent with the temporal dynamics of vigilance followed by avoidance in particular. That is, at Lag 1, anxious individuals will still be attending to the negative distractor, whereas by Lag 2 they have shifted their attention to the opposite stream to avoid it, thus resulting in the deficit in processing targets appearing in the same stream as the distractor (i.e., the pattern characteristic of spatially localized emotion-induced blindness). Thus, the temporal dynamics of spatially-localized emotion-induced blindness also support the vigilance-avoidance explanation for it.

In addition, work using event-related potentials (ERPs) suggests that the attentional blink and emotion-induced blindness have shared neural mechanisms (Kennedy et al., 2014; J. MacLeod et al., 2017). The attentional blink is typically ascribed to nonspatially specific mechanisms such as a central attentional bottleneck or resource sharing (e.g, Chun & Potter, 1995; Dell'Acqua et al., 2015; Martens & Wyble, 2010; Shapiro et al., 2006). Therefore, the evidence of shared mechanisms between the two further bolsters the notion that emotion-induced blindness may not require a spatially-specific mechanism to account for it. However, it should be noted that some attentional blink models do incorporate spatial components (Wyble & Swan, 2015).

At first blush, one potential challenge to this line of reasoning that a common attentional bottleneck underlies both the attentional blink and emotion-induced blindness is Lag-1 sparing. Lag-1 sparing is a phenomenon identified within the attentional blink literature, whereby when the second target immediately follows the first target (i.e., at Lag 1), the perception of the second target is quite high and relatively immune to the blink, which commences from Lag 2 onwards (e.g., Chun & Potter, 1995). Lag-1 sparing is not always observed and can depend on a variety of factors (e.g., Hommel & Akyurek, 2005; Livesey & Harris, 2011; Olivers & Meeter, 2008; Visser et al., 2009). However, Lag-1 sparing has typically not been observed at all in emotion-induced blindness (Kennedy & Most, 2015b; Most & Jungé, 2008). Does this mean that they reflect distinct underlying mechanisms? The short answer is no, because Lag-1 sparing does not differentiate the attentional blink from emotion-induced blindness. Instead, it is dependent on the stimuli used (e.g., pictorial versus alphanumeric), not whether they are used in the context of identifying two neutral targets in the stream or in the context of identifying a single target following an emotionally salient distractor (Huang et al., 2008; Livesey & Harris, 2011).

To summarize, the two-stage model (Chun & Potter, 1995) is an influential model for explaining effects in RSVP streams, which can account for emotion-induced blindness. In contrast, another theoretical account that has been offered for emotion-induced blindness espouses a spatiotemporally localized deficit (Most & Wang, 2011). This model was motivated to explain spatially specific effects in dual-stream emotion-induced blindness studies. However, this model has been challenged by findings suggesting that apparent evidence for spatial specificity to emotion-induced blindness following negative distractors could instead reflect vigilance avoidance, and therefore emotion-induced blindness does not necessarily require a spatially specific explanation (Proud et al., 2020). Furthermore, emotion-induced blindness following positive images has been found to occur in both streams (i.e., not spatially localized), which is consistent with a central-locus bottleneck, rather than an earlier spatial-specific mechanism. Consistent with this, emotion-induced blindness appears to share much in common with the attentional blink (Kennedy et al., 2014; MacLeod et al., 2017), for which the dominant explanations are nonspatial in nature (Martens & Wyble, 2010).

## Directions for future research

There are many important research questions that emotion-induced blindness can be used to address. Here, we identify several major themes, which we believe are both theoretically substantive, and have significant potential practical applications to understanding and ultimately reducing people’s heightened experiences of negative affect. First, we discuss how emotion-induced blindness can be used to adjudicate between major competing theoretical models of how emotion influences attention. Next, we discuss the potential for emotion-induced blindness to provide insight into competing theoretical models of how negative affect influences attentional prioritization, but we then identify how first the inconsistencies in the literature need to be addressed. We explain what we believe to be the root cause of the inconsistent findings regarding individual differences in emotion-induced blindness and discuss ways to rectify this. Finally, we identify how the paradigm can be used to assess questions beyond emotion per se, such as the attentional prioritization of self-relevant material.

The emotion-induced blindness literature has clearly shown that when emotionally salient stimuli receive attentional priority, they induce a temporary period of functional blindness during which other stimuli are less likely to be processed. However, there remain two quite distinct theoretical models to explain this effect—one espouses a spatially localized impairment, whereas the other attributes the blindness to a central, nonspatial attentional bottleneck. The finding of spatially localized deficits in dual-stream emotion-induced blindness following negative distractors led to the proposal of a spatially specific mechanism (Most & Wang, 2011), however, a more recent study suggests that this pattern only occurs for those with high levels of anxiety, consistent with attentional avoidance of the stream that contained the negative distractor, thus impairing perception of the target when it does occur in the same stream as the distractor (Proud et al., 2020). This vigilance-avoidance pattern could thus make it appear that there is a spatially specific deficit, when in fact it is just a consequence of anxious individuals avoiding the stream that contained a confronting item. Further consistent with this vigilance-avoidance idea, positive images were not found to induce spatially specific emotion-induced blindness in any individuals (Proud et al., 2020). However, this challenge to the spatially specific model is a single study, which leaves open some counterexplanations. For example, it is possible that there is a spatially specific deficit, but it is selective to negative stimuli and most pronounced in those with high levels of anxiety. Therefore, further testing is required to adjudicate between these theoretical accounts. For example, eye tracking may be particularly useful to determine whether there are eye-movement patterns consistent with individuals moving their attention away from the stream that negative distractor appeared in, consistent with vigilance avoidance rather than a true perceptual deficit. Alternatively, if on a trial a second negative emotionally salient distractor was presented in the opposite stream (opposite to the first negative emotionally salient distractor) soon after it, then the two different accounts would make different predictions for what would happen for subsequent target perception. According to the spatially localized deficit, both streams should be suffering a spatially localized deficit, and thus perception of a target should be poorer in either stream. In contrast, according to vigilance avoidance, the second presentation of the negative emotionally salient distractor should trigger avoidance of the second-distractor stream and thus reengagement with the first-distractor stream, thus leading to unimpaired perception of targets in the first stream.

Emotion-induced blindness can also be productively used to determine the relative predictive value of the two major ways of conceptualizing emotion—categorical versus dimensional approaches—to understanding the influence of emotion on attention. For example, do fear-inducing and disgust-inducing images produce different magnitudes of emotion-induced blindness, even when they are matched for their degree of negative valence and their arousal? If so, then this would indicate that categorical approaches to emotion explain variance in attention that the dimensional approach does not account for. In doing so, it is critical that such studies ensure that stimuli are matched on the dimensions of valence and arousal, via explicit ratings from the specific participants that provide behavioral data. In other aspects of the emotion-attention literature this has not been heeded, leading to considerable confusion. For example, there is ongoing debate about the extent to which motivational intensity is a dimension that is meaningfully dissociable from the dimensions of valence and arousal (Campbell et al., 2021; Kaczmarek et al., 2021). Proponents of the motivational intensity model of emotion–cognition interactions have often used stimuli in different conditions that they claim differ with respect to their motivational intensity. When differential patterns of performance on attentional tasks result from these conditions, they argue that they reflect motivational intensity, rather than valence (e.g., Harmon-Jones et al., 2012). However, in multiple studies, there was explicitly a significant difference in the *valence* ratings between the purportedly high- and low-motivational intensity stimuli. Despite this, the behavioral effects are attributed to motivational intensity (e.g., Gable & Harmon-Jones, 2008, 2010). We urge researchers to avoid such confounded designs here. Ensure that stimuli that come from different categories (e.g., fear versus disgust) are *matched* with respect to their degree of rated valence and arousal, before assessing their role in explaining emotion-induced blindness.

Further, in the literature on individual differences in trait anxiety, there are multiple competing theoretical accounts for how trait anxiety influences attentional allocation and control. It has long been theorized that anxiety is associated with an attentional bias toward negative or threatening stimulus materials (e.g., Bar-Haim et al., 2007; Delchau et al., 2022; MacLeod et al., 1986, 2019). In contrast, others predict that anxiety is associated with poor attentional control in general, not limited to in the presence of threatening stimuli (Berggren & Derakshan, 2013; Bishop, 2009; Eysenck et al., 2007; Moran, 2016; Shi et al., 2019). Emotion-induced blindness occurs for both positive and negative stimuli, relative to neutral (Most et al., 2005, 2007; Proud et al., 2020). However, when investigating individual differences in emotion induced blindness, such as whether individuals prone to negative affect experience heightened effects, researchers often just employ negative (versus neutral) stimuli. This is a missed opportunity, because it is only for the positive condition that these two competing models make differential predictions. Therefore, future research should use both the negative and positive distractors to differentiate between these competing models. If anxiety is associated with a negative-specific bias, then anxious individuals should show selectively increased emotion-induced blindness following negative and not positive images. If anxiety is associated with general attentional control deficits, then anxious individuals should have difficulty inhibiting both types of salient distractors, and thus show exacerbated emotion-induced blindness following both positive and negative images.

However, before emotion-induced blindness can be used to test such individual difference-based questions, the inconsistencies of relationships between emotion-induced blindness and some of the individual difference variables that have been measured need to be addressed. We believe that these inconsistencies stem from two major sources, each of which is discussed in more detail below: (1) low measurement reliability, and (2) failure to account for important moderating individual difference variables.

It was first published in 1910 that the observed correlation between two variables is fundamentally constrained by the measurement reliability of each of the variables (Spearman, 1910). This means that a study may fail to observe a significant correlation between Variable A and Variable B, even if there truly is a correlation between the constructs that these variables operationalize, if one or both of the variables have poor measurement reliability. This fact is well understood in questionnaire-based individual difference and personality research. It is why measures of reliability are routinely reported, considered, and interpreted there. However, it is an equally important consideration when one or more of the variables derive from experimental measures—such as when assessing the relationship between emotion-induced blindness and anxiety. However, it is much rarer in studies using experimental paradigms to answer individual difference questions to see reliability explicitly considered at all. There have been some recent calls to rectify this and promote reporting and consideration of reliability more broadly (Goodhew & Edwards, 2019; Parsons et al., 2019), but it remains a widespread issue. We believe that low-measurement reliability lies at the heart of the inconsistent emotion-induced blindness individual-difference findings. Here, we explain why, and how to fix this.

First, however, it is important to be clear about some concepts that are sometimes confused. Measurement reliability is distinct from replicability, which is usually taken in this context to mean the consistency with which an effect is observed in a sample. Instead, measurement reliability can be thought of as the consistency with which the outcome measure rank-orders individuals. For example, if Person A has a larger emotion-induced blindness magnitude and Person B a smaller one at Time 1, will this remain so at Time 2? Emotion-induced blindness is highly replicable. However, there are several lines of evidence which cast doubt on its measurement reliability.

This discussion can be understood in the context of seminal work demonstrating the inherent tension between replicability and reliability (Hedge et al., 2018). Hedge et al. (2018) showed that paradigms such as the Stroop effect, which are highly replicable, have poor reliability, because the attribute that promotes their replicability (i.e., low between-participant variability) directly compromises measurement reliability. That is, if most individuals succumb to an effect and do so to similar extents, then there is minimal between-participant variability and the measure will have low reliability. Instead, a large (and consistent) spread of individuals in their scores promotes reliability (Hedge et al., 2018). Given the robustness of emotion-induced blindness as an experimental effect, we believe that emotion-induced blindness in its current form may also have low between-participant variability, and thus low reliability.

To our knowledge, only one published study has explicitly measured the reliability of emotion-induced blindness, and it indicated that the intraclass correlation coefficient (ICC) of the emotion-induced blindness difference score was modest: .42 at Lag 2 (Onie & Most, 2017). While the absolute accuracy scores for accuracy in the emotionally salient condition had greater reliability, absolute scores conflate about emotional and nonemotional sources of variance (e.g., general processing speed), the effects of which are removed from a measure only when performance is compared between two conditions where these are held constant (i.e., a difference score between the emotionally salient and emotionally neutral conditions is used to quantify the effect of emotional salience on perception; Goodhew & Edwards, 2019). Therefore, the difference score is the most valid measure of the selective influence of emotion on attention.

Onie and Most (2017) found that the measurement reliability for the emotion-induced blindness difference score was greater than that for the dot-probe measure used in that same study, but still a way off what would be considered adequate reliability for a questionnaire measure. It is important to note that reliability is not a fixed property of an experimental technique but will depend on a number of factors, such as the stimuli used for emotion-induced blindness, and the participants and their characteristics, and of course the interaction of these factors. If a large corpus of studies on emotion-induced blindness has reported the measurement reliability of emotion-induced blindness, then it might be possible to estimate where studies with particular designs might fall with respect to reliability. With just a single one, however, it is possible, likely even that there is considerable variability around this, such that some studies may have prohibitively low measurement reliability that preclude the possibility of obtaining a correlation with another measure, even if there is one to be found. We believe that this explains the inconsistencies observed in the literature on individual differences in emotion-induced blindness.

So, how to fix this? Step 1 is for measurement reliability to be reported in emotion-induced blindness studies (and, indeed, in all individual difference studies, including those that use experimental measures derived from cognitive psychology; Goodhew & Edwards, 2019). Step 2 is to improve the measurement reliability of emotion-induced blindness. This is, of course, nontrivial and will require innovation and repeated testing. However, as a preliminary first step, we believe that increasing between-participant variation may occur when the potency of emotion-induced blindness is scaled back. That is, we believe that, at present, emotion-induced blindness may be a victim of its own success as a robust experimental technique—the effect is so strong that the vast majority of participants experience it to a large extent. Versions of emotion-induced blindness that scale back the potency of the paradigm may increase between-participant variation, such that different participants experience it to different degrees, and some not even at all. For example, it is notable that emotion-induced blindness following words appears less potent than that following pictures, suggesting it might be a more reliable version of emotion-induced blindness. Step 3 is to reassess the relationship between emotion-induced blindness with these more reliable versions of the paradigm and previously tested individual difference variables such as negative affect and attentional control.

We also believe that some of the observed inconsistencies in relationships between emotion-induced blindness and individual difference variables, like negative affect, may stem from a failure to consider other important individual difference variables. In particular, we believe that *empathy* may be an important moderator of emotion-induced blindness, especially for particular types of eliciting images. An image of another person being attacked, or being pleasured, is not isomorphic with being attacked, or being pleasured oneself. Instead, it requires the psychological process of empathy, to feel with or for others, for these stimuli to have an effect. Therefore, failure to account for individual differences in empathy may lead to inconsistent relationships between emotion-induced blindness and other individual difference variables.

There are two main ways to address variation in empathy affecting emotion-induced blindness if empathy is not of interest itself. One solution is to use stimuli with learned associations. A stimulus that has been associated with reward or punishment for a participant themselves does not require the participant to have empathy for it to have emotional salience. The other approach is simply to measure empathy and control for it statistically. In considering empathy via this approach, we urge researchers to consider the fundamental distinction between cognitive empathy (understanding someone else’s thoughts, feelings, and perspective, akin to theory of mind), and affective empathy (feeling what someone else is feeling), as there is compelling evidence that cognitive and affective empathy call upon distinct mechanisms (Cox et al., 2012; Eres et al., 2015; Goodhew & Edwards, 2021; Kanske et al., 2015; Preckel et al., 2018; Shamay-Tsoory et al., 2009; Tholen et al., 2020). It may also be important to consider the motivational dimension of empathic concern (i.e., extent to which a person is motivated to promote the well-being and alleviate the suffering of others (Weisz & Cikara, 2021). Indeed, attentional control is thought to be implicated in emotion-induced blindness, and it has *qualitatively* different relationships with different aspects of empathy (Goodhew & Edwards, 2021, 2022b). In light of the distinction between affective and cognitive empathy, it is interesting that individuals scoring high in psychopathy have been reported to have reduced emotion-induced blindness (Kimonis et al., 2020), since such individuals are typically thought to have impaired affective empathy but intact cognitive empathy (Lamm et al., 2016). This suggests that affective empathy might be particularly important in understanding emotion-induced blindness, such that individuals with higher levels of it may experience exacerbated emotion-induced blindness. In addition, cognitive empathy is positively associated with attentional control (Goodhew & Edwards, 2021), and attentional control appears to reduce emotion-induced blindness (Peers & Lawrence, 2009), so there is reason to think that cognitive empathy may provide a protective effect against emotion-induced blindness. Altogether, it is useful to consider individual differences in both the cognitive and affective components of empathy, to clarify the relationships between other individual difference variables and emotion-induced blindness.

Further, consistencies in the way that attentional control relates to emotion-induced blindness may stem from inadequate operationalization of the construct. In particular, to date attentional control has been treated as unitary concept in the emotion-induced blindness literature, but there is evidence that the focusing and switching components of attentional control have qualitatively different relationships with other variables (Goodhew & Edwards, 2021; Judah et al., 2014). Qualitatively different relationships between emotion-induced blindness and the two separate components could distort apparent relationships between emotion-induced blindness and attentional control. For example, if the focusing and switching components had opposite-direction relationships with emotion-induced blindness, then this could cancel out to misleadingly appear as a null relationship. Therefore, it is crucial to consider this distinction between assessing relationships between attentional control and emotion-induced blindness.

Finally, we suggest that one interesting avenue forward is to establish whether emotion-induced blindness is limited to stimuli that are salient for *emotional* reasons, versus whether it generalizes to other forms of salience. For example, it has been shown that participants can quickly learn to associate geometric shapes with *self* (versus friend or stranger), such that this learning influences their response efficiency, called the self-prioritization effect (Sui et al., 2012; Sui & Rotshtein, 2019). Do such self-relevant stimuli produce emotion-induced blindness? Addressing such questions will provide valuable information about the extent to which self-relevant stimuli command attentional priority in the way that emotionally salient ones do.

## Conclusion

In conclusion, emotion-induced blindness is a robust effect that demonstrates the potent influence of emotional-salience on attention and perception. It has yielded important insights about the relationship between emotion and attention and holds the promise of productively contributing to research questions of both theoretical and practical importance. Here, we have outlined several innovative directions for future research that we believe will help the field to arrive at a deeper understanding of the nexus between two fundamental aspects of human experience—the experience of emotion, and the influence attentional selection shaping our perception of the world around us.

## Data Availability

N/A (because this is a qualitative review piece)

## Footnotes

1 At times in the literature the term *emotional* is used to describe such stimuli. However, we believe that the term *emotional salience* better reflects the properties of the stimuli and how they are perceived.

2 Note that attentional orienting to emotionally salient stimuli under such conditions may still be goal driven, just driven by goals that the participant brings into the laboratory (e.g., to attend to stimuli relevant to self-preservation and reproduction), rather than any goals induced by the experimental instructions (e.g., attend to a target letter).

3 Of course, one could argue that emotionally salient distractors are *more different* from the fillers than the neutral distractors are, and this therefore could be the cause of the more pronounced target perception deficit following emotionally salient distractors. However, as discussed below, emotion-induced blindness is observed even with stimuli that are completely matched on the physical dimension, and differ only in their learned emotional salience. This means that there is clearly variance in emotion-induced blindness attributable to emotional salience beyond physical salience. Further, the magnitude of emotion-induced blindness scales according to participants’ ratings of the emotional aspects of images, indicating that the deficit is emotional rather than physical in nature (Onie & Most, 2021).

4 In the literature, sometimes the term *emotion-attentional blink* is used interchangeably with *emotion-induced blindness*. Here, however, we reserve the term *emotion-induced blindness* for single-target RSVP streams and *attentional blink* (emotional or otherwise) for two-target RSVP streams.

5 We note that visual angle is a more useful metric for comparisons across studies than absolute stimulus size. Although not reported, if we assume a viewing distance of 60 cm in the original study, then this would correspond to about 14° × 10°. In our own work and pilot testing, we have found emotion-induced blindness to be robust across a range of stimulus sizes, except when the images are particularly small.

6 Valence is one of the core dimensions of human emotion, along with arousal. Valence refers to the pleasantness of the emotion, which varies from unpleasant to pleasant with neutral in between. Negative valence = unpleasant whereas positive valence = pleasant. The role of valence versus arousal in emotion-induced blindness is discussed further in a later section of this paper.

7 While the difference score measure accounts for individual differences in generic perceptual processing speed unrelated to emotion that influences raw scores, it can still be important to consider raw scores. For example, if a larger emotion-induced blindness magnitude in a condition was obtained by changes in accuracy only following the emotionally neutral distractors, then this would require a different interpretation to one obtained by changes in accuracy following the emotionally salient distractors.
